# The Evolution of Aggregative Multicellularity and Cell–Cell Communication in the Dictyostelia

**DOI:** 10.1016/j.jmb.2015.08.008

**Published:** 2015-11-20

**Authors:** Qingyou Du, Yoshinori Kawabe, Christina Schilde, Zhi-hui Chen, Pauline Schaap

**Affiliations:** College of Life Sciences, University of Dundee, Dundee DD1 4HN, United Kingdom

**Keywords:** evolution of multicellularity, encystation, sporulation, cyclic nucleotide, dual component signalling

## Abstract

Aggregative multicellularity, resulting in formation of a spore-bearing fruiting body, evolved at least six times independently amongst both eukaryotes and prokaryotes. Amongst eukaryotes, this form of multicellularity is mainly studied in the social amoeba *Dictyostelium discoideum*. In this review, we summarise trends in the evolution of cell-type specialisation and behavioural complexity in the four major groups of Dictyostelia. We describe the cell–cell communication systems that control the developmental programme of *D*. *discoideum*, highlighting the central role of cAMP in the regulation of cell movement and cell differentiation. Comparative genomic studies showed that the proteins involved in cAMP signalling are deeply conserved across Dictyostelia and their unicellular amoebozoan ancestors. Comparative functional analysis revealed that cAMP signalling in *D*. *discoideum* originated from a second messenger role in amoebozoan encystation. We highlight some molecular changes in cAMP signalling genes that were responsible for the novel roles of cAMP in multicellular development.

## Highlights

• Many eukaryotes and prokaryotes aggregate to form a community in which cells together construct a fruiting body.
• Quorum sensing, two-component signalling and signalling mediated by the cyclic nucleotides cAMP and c-di-GMP play major roles in the social behaviour of prokaryotes.
• Similar signalling mechanisms also regulate aggregation and cell-type specialisation during fruiting body formation in the eukaryote *Dictyostelium discoideum*.
• Comparative genomic and gene functional analysis revealed that the cAMP and two-component signalling mechanisms that control *Dictyostelium* development originated from a stress response in the unicellular ancestors.

## Graphical abstract

**Figure:**
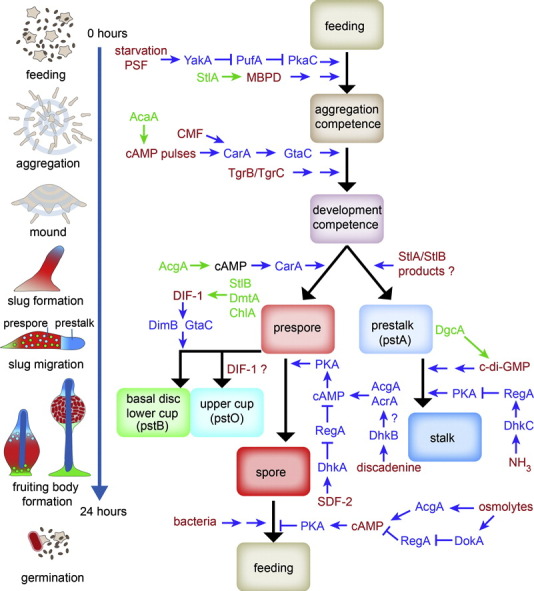

## Aggregative Multicellularity in Eukaryotes

**Fig. 1:**
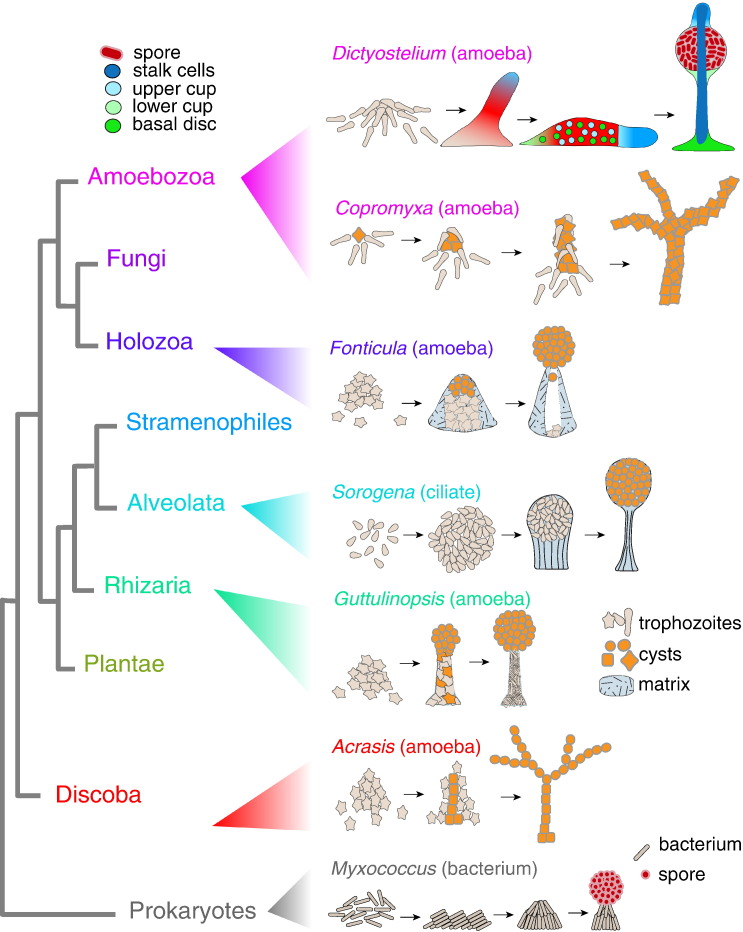
Organisms with aggregative multicellularity. Unicellular eukaryotes mostly have a simple life cycle consisting of a trophozoite feeding stage and a dormant cyst stage. In most eukaryote divisions and in prokaryotes, multicellular forms that aerially lift dormant spores or cysts in fruiting bodies evolved (sorocarps). Starving *Myxococcus* bacteria aggregate to form fruiting bodies, in which part of the rod-shaped bacteria differentiate into spherical spores. *Acrasis* amoebae aggregate and start encysting at the base of the structure. Other amoebae crawl to the top, rearrange themselves into chains and then encyst. *Guttulinopsis* amoebae construct a column surrounded by an elastic sheath. Some amoebae move to the top and differentiate as spores, whilst the remaining amoebae gradually disintegrate. The ciliate *Sorogena* aggregates by cell adhesion to form a mound encased in a mucous sheath. The sheath contracts to form a stalk that lift up the cells, which then encyst. *Fonticula* amoebae deposit a cone-shaped matrix around the aggregates. The amoebae then differentiate into spores and are expulsed through the apex. *Copromyxa* amoebae move towards a few encysted founder cells. Once aggregated, cells crawl on top of existing cysts and then encyst themselves. *Dictyostelium* aggregates first form migrating slugs. Inside the slug, the cells differentiate into precursors for the spore, stalk, basal disc and upper and lower cup cells. During fruiting formation, the precursor cell types move to their appropriate locations and complete the differentiation process.

We are much more familiar with large multicellular organisms in the eukaryote domain, such as animals, plants and fungi, than with the unicellular organisms from which they evolved. The genetic diversity of eukaryotes is nevertheless much larger than the combined diversity of the multicellular forms [1], [2]. The eukaryotes comprise an immense range of morphologically distinct unicellular organisms and, in addition to animals, plants and fungi, at least six examples of organisms that independently made the transition from unicellularity to multicellularity (Fig. 1). Because these multicellular forms are rarely larger than a few centimetres, they are not commonly known.

Almost all multicellular organisms pass through a unicellular stage at least once in their life cycle. This single cell then divides repeatedly to generate the multicellular form. In animals, plants and fungi, the offspring of the first cell, a fertilised egg or a spore, remains attached to each other. However, in most other multicellular organisms, the cells disperse after cell division to maximise their access to food. They only come together again, when starved or otherwise stressed, to build a multicellular fruiting body or sorocarp with resilient cysts or spores.

This life cycle, termed alternatively aggregative or sorocarpic multicellularity, is not unique to eukaryotes and is also used by the myxobacteria in prokaryotes [3]. Eukaryotes that display aggregative multicellularity are *Acrasis* in the division Discoba [1], [4], *Fonticula alba* in Holozoa [5], *Guttulinopsis* spp. in Rhizaria [6], *Sorogena stoianovitchae* in Alveolata [7], [8] and *Copromyxa* and Dictyostelia spp. in Amoebozoa [9], [10].

For some genera, such as *Acrasis* and *Copromyxa*, the starving cells crawl on top of each other and differentiate into spores or cysts (Fig. 1). In others, such as *Fonticula* and *Sorogena*, the aggregated cells first deposit a structured extracellular matrix to support the spore mass. *Guttulinopsis* spp. show a primitive form of cell specialisation. Amoebas destined to become spores crawl to the top of the aggregate, whilst those that are left behind synthesise fibrous material to support the spores and then decay [11], [12]. Amongst aggregating eukaryotes, the Dictyostelia display the most sophisticated form of multicellularity, with a freely moving “slug” stage and up to five different cell types [13].

## Evolution of Multicellular Complexity in Dictyostelia

The Dictyostelia are the largest group of eukaryotes with aggregative multicellularity, with around 150 known species. Molecular phylogenetic inference subdivides species into four major and two or three minor groups [14], [15]. Groups 1–3 consist mostly of species that form multiple small fruiting bodies from a single aggregate and/or fruiting bodies with multiple side branches. The fruiting bodies mainly form directly after aggregation, with no or little slug migration. The process of cell differentiation is relatively simple. After aggregation, the cells differentiate first into prespore cells and only cells that have reached the tip of the structure then transdifferentiate into stalk cells [16], [17]. The Acytostelids, which form clade 2A of group 2, do not form a cellular stalk. Here the prespore cells express both spore- and stalk-specific markers and collectively construct a central cellulose stalk tube. They next move up this tube and mature into spores [18].

In addition to forming multicellular fruiting bodies with spores, many species in groups 1–3 can still encapsulate individually as cysts, and have thus retained the survival strategy of their unicellular ancestors. The ability to encyst is lost in group 4, which additionally shows a pronounced increase in multicellular complexity. Species in group 4 generally form a large unbranched fruiting body from a single aggregate. Extensive migration of the sorogen or “slug” often precedes fruiting body formation, and cell differentiation is highly regulated. In the slugs, the amoebas differentiate into prespore and prestalk cells in proportions that reflect the ratio of spore to stalk cells in the fruiting body. The prestalk and prespore cells are at first intermixed, but they later sort out to form a well-defined anterior–posterior prestalk/prespore pattern. Additional cell types differentiate in the posterior, which will later form a basal disc to support the stalk and an upper cup and a lower cup to support the spore head. Group 4 is also unique in using cAMP as the chemoattractant for aggregation. In groups 1–3, the dipeptide glorin is most commonly used and more rarely folate, pterin or unknown compounds [16], [17].

In addition to encystation and fruiting body formation, Dictyostelia also have a sexual life cycle, where amoebas of opposite mating type fuse to form a zygote. The zygote then attracts and cannibalises cells of the same species and uses their contents to build a very resilient multilayered cell wall [19]. Species scattered over all four groups form these zygotic cysts or macrocysts, suggesting that this is an ancient survival strategy of Dictyostelia [15].

## Cell–Cell Signalling during the *Dictyostelium discoideum* Life Cycle

### Quorum sensing regulates the growth to development transition

The mechanisms that enable and regulate the multicellular life cycle of Dictyostelia were investigated almost exclusively in the model organism *Dictyostelium discoideum*, a member of group 4. Its popularity is due to the fact that procedures for genetic transformation were first developed for this species [20], soon to be followed by a wide range of molecular genetic and cell biological methodologies.

**Fig. 2:**
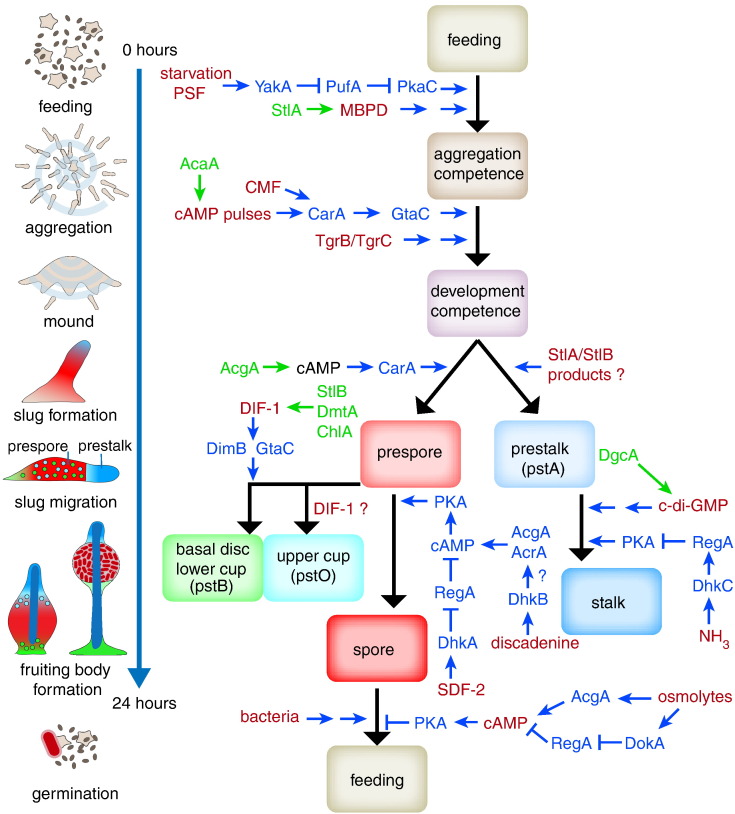
Cell signalling during *Dictyostelium* development. (a) The asexual life cycle of *D*. *discoideum*. (b) Cell signalling mechanisms. The schematic shows the signals (in red) that control the life cycle transitions and the differentiation of amoebae in spores and somatic cell types. The enzymes that synthesise secreted signal molecules are shown in green text, and proteins and small molecules involved in the intracellular signal transduction pathway are in blue text. Blue arrows and t-crosses denote stimulatory and inhibitory effects, respectively. Double blue arrows signify that no components of the signal transduction pathway are known. All pathways are described in detail in the main text. Abbreviations: PSF: prestarvation factor; MPBD: 4-methyl-5-pentylbenzene-1,3-diol; cAMP: 3′-5′-cyclic adenosine monophosphate; CMF: conditioned medium factor; Tgr: transmembrane, IPT, IG, E-set, repeat protein; DIF-1: differentiation inducing factor 1; c-di-GMP: 3′,5′-cyclic diguanylic acid; NH_3_: ammonia; SDF-2: spore differentiation factor 2; StlA: Steely A; AcaA: adenylate cyclase A; StlB: Steely B; AcgA: adenylate cyclase G; DmtA: des-methyl-DIF-1 methyltransferase; ChlA: chlorination A; DgcA: diguanylate cyclase A; YakA: DYRK family protein kinase; PufA: pumilio RNA-binding protein; PkaC: cAMP-dependent protein kinase, catalytic subunit; CarA: cAMP receptor 1; GtaC: GATA-binding transcription factor C; DimB: transcription factor DIF-insensitive mutant B; RegA: cAMP phosphodiesterase RegA; AcrA: adenylate cyclase R DhkA: histidine phosphatase A; DhkB: histidine kinase B; DhkC: histidine kinase C; DokA: osmosensing histidine phosphatase.

Starvation is the major trigger for entry into aggregative development, but the process is fine-tuned by the ability of amoebas to monitor their own cell density relative to that of their bacterial prey (Fig. 2). The growing cells secrete a glycoprotein, PSF (*p*re*s*tarvation *f*actor) at a constant rate [21], which acts as a quorum sensing factor coordinating gene expression relative to cell density [22]. A combination of low bacterial density and high PSF induces expression of the protein kinase YakA [23], which inhibits binding of the translational repressor PufA to the 3′-end of the catalytic subunit of cAMP-dependent protein kinase (*PkaC*) [24]. PkaC is consequently translated and proceeds to induce expression of genes that are required for aggregation, such as the cAMP receptor *carA*, the adenylate cyclase *acaA* and the extracellular cAMP phosphodiesterase *pdsA* [25]. In addition to PSF, the starving cells secrete a polyketide MPBD (4-*m*ethyl-5-*p*entyl*b*enzene-1,3-*d*iol), which is also required for rapid expression of aggregation genes [26], and a protein, CMF (*c*onditioned *m*edium *f*actor), which is essential for CarA-mediated signal transduction [27]. MPBD is synthesised by the polyketide synthase StlA and plays a second role in spore maturation in later development.

### cAMP oscillations and cell–cell interactions prepare cells for post-aggregative development

CarA, AcaA and PdsA are key components of the network that autonomously generates pulses of cAMP [28]. These pulses are initially secreted by a few starving cells and propagate as waves through the cell population [29]. Cells move chemotactically towards a local cAMP source and collect into mounds. The utmost tip of the mound continues to emit cAMP pulses, which, by attracting cells from underneath, causes the cell mass to form the cylindrical sorogen or slug and later the fruiting body [30].

In addition to inducing chemotaxis, the cAMP pulses upregulate expression of genes that are required during and after aggregation by acting on the transcription factor GtaC [31], [32]. These genes are *carA*, *acaA*, *pkaR* and *regA* and the cell adhesion genes *csaA*, *tgrB1* and *tgrC1*. TgrB1 and TgrC1 are members of a family of transmembrane proteins with highly polymorphic extracellular domains. Heterophilic interactions between compatible TgrB and TgrC proteins induce competence for post-aggregative cell differentiation (Fig. 2), and additionally serve the purpose of kin recognition, preventing non-related strains from participating in the same fruiting structure and forming an unfair share of spores compared to stalk cells [33], [34], [35].

### cAMP, DIF-1 and c-di-GMP induce cell-type specialisation

After aggregation, a second adenylate cyclase, AcgA, is translationally upregulated in the posterior of the slug, where increased cAMP levels induce the differentiation of prespore cells [36], [37]. The prespore cells start to synthesise spore wall materials in Golgi-derived vesicles and additionally express the enzymes StlB, DmtA and ChlA that synthesise the chlorinated cyclohexanone DIF-1 [38], [39], [40]. DIF-1 synthesis causes differentiation of other posterior cells into prestalk O (pstO) cells, which later form the upper cup of the fruiting body, and into prestalk B (pstB) cells, which will form the lower cup and basal disc. A polyketide produced by either StlB or StlA, which is neither DIF-1 nor MPBD, is required for expression of genes at the anterior of the prestalk region. However, StlA and/or StlB are not required for the differentiation of stalk cells [39], [41].

The signal for stalk cell differentiation is c-di-GMP, which is synthesised by diguanylate cyclase A in both prestalk and stalk cells [42]. Diguanylate cyclases were previously only found in prokaryotes, where c-di-GMP is the intracellular intermediate for a range of stimuli that induce biofilm formation and other cellular responses [43].

### Sensor histidine kinase and PKA-mediated signalling controls spore and stalk cell maturation

Fruiting bodies are formed by organised amoeboid movement, but the amoebas are meanwhilst becoming immobilised by cell walls as they are differentiating into spore and stalk cells. Several pathways acting in parallel therefore tightly control terminal differentiation. These pathways ultimately converge on the activation of PKA by cAMP. PKA activity is essential for both stalk and spore maturation and additionally prevents the germination of spores under conditions unfavourable for growth [44], [45], [46], [47]. AcgA and a third adenylate cyclase, AcrA, synthesise cAMP at this stage [48], but cAMP hydrolysis by the cytosolic phosphodiesterase RegA plays the most dominant role in regulation of PKA activity. The phosphodiesterase activity of RegA is activated by phosphorylation of its N-terminal response regulator domain by sensor histidine kinases (SHKs) [49], [50]. Most of the signals that control spore and stalk differentiation act either on SHKs to phosphorylate and activate RegA or on sensor histidine phosphatases (SHPs) to dephosphorylate and thereby inhibit RegA.

Stalk cell differentiation is under negative regulation of ammonia, which is produced in large quantities by protein degradation in the starving cells [51]. Ammonia activates the SHK DhkC, thereby activating RegA and inhibiting PKA [52]. Ammonia is lost from the aerially projecting tip of the early fruiting body, thus inactivating RegA and lifting PKA inhibition. Spore maturation requires release of the protein AcbA by prespore cells, which is cleaved by prestalk cells to yield the peptide SDF-2 [53]. SDF-2 in turn activates the SHP DhkA on prespore cells, which dephosphorylates RegA and thereby activates PKA [54].

Cells in fruiting bodies also secrete an adenine analogue, discadenine, which acts both to stimulate spore maturation and to inhibit spore germination. Genetic evidence indicates that the effects of discadenine are mediated by the SHK DhkB and AcrA [55], [56]. AcrA has two response regulator domains, but neither of these is required for AcrA activity [57]. It is therefore not yet clear how DhkB activates AcrA. A third factor contributing to spore maturation and preventing spore germination is the ambient high osmolarity of the spore head, which induces PKA activation by two different pathways. Firstly, high osmolarity is perceived by the extracellular osmosensor of AcgA, activating cAMP synthesis. Secondly, high osmolarity activates the SHP DokA, which in turn inactivates RegA [46], [58], [59]. A surprisingly large number of seemingly redundant pathways control the maturation and germination of spores (see also Ref. [60]). This likely reflects that the multicellular life cycle of Dictyostelia is a survival strategy that culminates into the differentiation of viable dispersible spores, which should only germinate when food is plentiful.

### Prokaryote-type signalling is prevalent in *Dictyostelium* development

Several signal molecules with major functions in *Dictyostelium* development, such as cAMP and c-di-GMP, are also widely used in the prokaryote domain [61], with particularly c-di-GMP playing a major role in the association of bacteria in multicellular communities [43]. Two-component signalling systems, which consist minimally of a sensor histidine kinase/phosphatase and response regulator, represent the major mechanism for environmental sensing in prokaryotes [62] and are particularly important in controlling spore and stalk encapsulation in *Dictyostelium*. In addition, synchronisation of gene expression by quorum sensing is of crucial importance in early development of both *Dictyostelium* and myxobacteria [63]. One reason for the use of prokaryote-type signalling in Dictyostelia could be that these signalling mechanisms are particularly suited for the *Dictyostelium* life style, another that the *Dictyostelium* mechanisms directly evolved from prokaryote counterparts. More insight in the extent to which *D*. *discoideum* signalling is conserved within the Dictyostelia as a group and more deeply in their amoebozoan ancestors and other eukaryotes is required to resolve this question.

## Evolutionary Reconstruction of Developmental Signalling in Dictyostelia

### Comparative genomics

The genetic diversity within Dictyostelia indicates that they evolved from the last common ancestor about 0.6 billion years ago [64]. All life forms are the product of selection acting on random mutations to favour reproduction in a particular niche. This implies that there is no logic to a complex regulatory process other than the order in which its component parts evolved from an earlier state. To understand why a particular process is built up the way it is, it is essential to first reconstruct its ancestral state and next retrace how genetic change altered the process in derived lineages.

Comparative analysis of genomes that span the genetic diversity of the group of interest can yield information on the core set of genes that are present in all members and specific changes that occurred in different lineages [65]. By correlating genetic change with phenotypic innovations and testing putative causal relationship by gene replacement, it is possible to reconstruct how developmental control mechanisms evolved and generated increasing phenotypic complexity.

The genomes of species that represent all major groups of Dictyostelia have been sequenced and assembled to a high level of completion [64], [66] (G. Gloeckner and P. Schaap, unpublished results). Draft genome sequences of additional group 4 and group 2 species are also available [67], [68], as well as the genome sequence of the unicellular amoebozoan *Acanthamoeba castellanii* [69]. The Dictyostelid genomes are all 31–34 Mb in size, with the exception of the *Dictyostelium lacteum* genome in group 2 with a size of 22 Mb. The *D*. *lacteum* genome has the same number of genes (~ 12,000) as the others but contains less intergenic sequence and introns. The genome of *Acanthamoeba* is with 45 Mb and 15,400 genes considerably larger than that of the Dictyostelia, indicating that the evolution of multicellularity in Dictyostelia did not require more genes.

Global analysis of gene families involved in cell signalling shows that, amongst Dictyostelia, group 4 has about 30% more G-protein-coupled receptors than groups 1–3. However, the numbers of genes encoding heterotrimeric and monomeric G-proteins, sensor histidine kinases and transcription factors are about the same [64]. Polyketide synthase genes are 3 to 10-fold reduced in groups 1 and 2 compared to group 4, with each group showing considerable gene gain and loss [64], [68]. *Acanthamoeba* has 30% less G-protein-coupled receptors than *D*. *discoideum* but 30% more protein kinases and three times the number of sensor histidine kinases. There is a large family of 67 adenylate cyclases in *Acanthamoeba*, which is not present in Dictyostelia and a single ortholog of *AcrA*. Strikingly, *Acanthamoeba* has metazoan-type tyrosine kinases, which are not present in Dictyostelia, and three times the number of proteins with SH2 domains that interact with phospho-tyrosines [69]. In sheer number of cell signalling genes, the strictly unicellular *Acanthamoeba* therefore exceeds *Dictyostelium*. This suggests that innovation of gene function is probably more important for the evolution of multicellularity than a mere gain in gene numbers.

### Comparative functional analysis—Genes involved in intracellular cAMP signalling

Genome comparisons provide very broad information on gene gain and loss. However, deeper and more targeted analysis of conservation and change in genes with known functions has thus far yielded the greatest insight into the evolution of developmental signalling in Dictyostelia. The most striking aspect of *Dictyostelium* development is the prominent role of cAMP (Fig. 2). As a secreted signal, it coordinates cell movement during aggregation and fruiting body formation and induces expression of aggregation genes and prespore genes. In a classical second messenger role, it mediates effects of many different stimuli that control the differentiation of spore and stalk cells and the germination of the spores. The proteins involved in the second messenger role of cAMP are the adenylate cyclases AcgA and AcrA; the cAMP phosphodiesterase RegA; the sensor histidine kinases DhkA, DhkB, DhkC and DokA; and the catalytic (C) and regulatory (R) subunits of PKA. *AcgA* and *acrA* are conserved in all Dictyostelid genomes, and *acrA*, *regA*, *pkaC* and *pkaR* are also present in *Acanthamoeba*. *DhkB*, *dhkC* and *dokA* are conserved throughout Dictyostelia, but there is no *dhkA* ortholog in group 1. None of *Dictyostelium* enzymes have clear orthologs amongst the 48 *Acanthamoeba* histidine kinases [64], [68], [69].

To assess whether conserved genes also have similar functions across Dictyostelia or even Amoebozoa, we analysed their roles by gene knockout in the group 2 species *Polysphondylium pallidum* or by pharmacological intervention with protein function in *Acanthamoeba castellanii*. As is the case in *D*. *discoideum*, disruption of *pkaC* in *P*. *pallidum* prevents entry into multicellular development, but *P*. *pallidum* amoebae also lose the ability to encyst [70], [71]. As described above, group 4 species, such as *D*. *discoideum*, have lost this ancestral survival strategy. In *D*. *discoideum*, the combined deletion of *AcrA* and *AcgA* prevents spore differentiation [37]. However, in *P*. *pallidum*, *acra*−*acga*− double mutants lose encystation but not spore differentiation. This is probably due to the presence of two additional *acaA* genes in *P*. *pallidum* of which one is expressed in prespore cells. Loss of RegA accelerates multicellular development in *D*. *discoideum* [49], and this is also the case in *P*. *pallidum*. However, *P*. *pallidum regA*− amoebae also encyst precociously, when sufficient food is still available [72]. A specific inhibitor of *A*. *castellanii* RegA also causes precocious encystation, and this is accompanied by elevated intracellular cAMP [72].

**Fig. 3:**
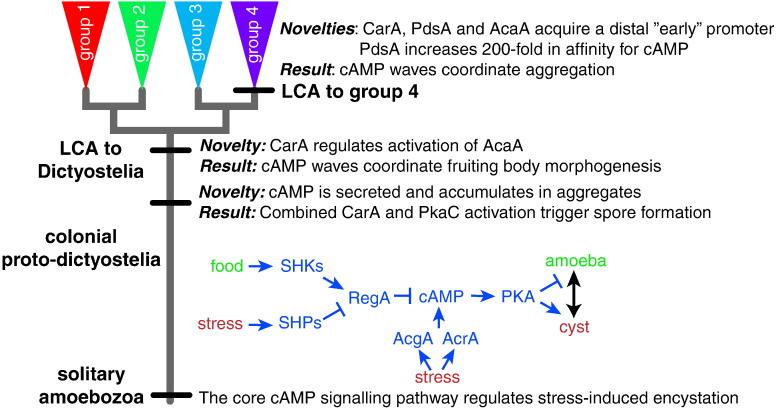
Hypothetical scenario for the evolution of developmental cAMP signalling in Dictyostelia. The cAMP signalling mechanisms that coordinate *Dictyostelium* development likely evolved from a core function of cAMP as intermediate for stress-induced encystation in the unicellular ancestor. In this role, stress acts on sensor histidine phosphatases to inhibit the cAMP phosphodiesterase RegA, allowing cAMP levels, produced by AcgA or AcrA, to increase and activate PKA, which subsequently induces encystation. The roles of secreted cAMP in induction of spore formation and in coordination of fruiting body morphogenesis evolved later, with the chemoattractant role of cAMP during aggregation only emerging in the last common ancestor (LCA) to group 4.

When combined, these studies show that cAMP acting on PKA has a core function in triggering encystation of single-celled amoebas in response to nutrient stress. In Dictyostelia, cAMP levels are negatively regulated by RegA and positively regulated by AcrA and AcgA (Fig. 3). In *Acanthamoeba*, RegA and probably AcrA have similar functions. In the course of Dictyostelid evolution, the roles of PKA, AcrA, AcgA and RegA were co-opted to additionally regulate the differentiation of spores and stalk cells. RegA, pkaC, pkaR, adenylate cyclases and a large family of sensor histidine kinases are also present in *Naegleria gruberi* [73], an unrelated amoeboflagellate from the division Discoba, the closest eukaryote relatives to prokaryotes [1]. Like most protozoa, *Naegleria* also encyst in response to stress. A role for cAMP in *Naegleria* encystation has yet to be demonstrated, but the conservation of the relevant cAMP signalling genes in this organism suggests that this is likely.

cAMP-mediated encystation and its regulation by sensor histidine kinases/phosphatases may therefore be very deeply conserved in eukaryotes. During regulation of encystation and cyst germination, sensor histidine kinases would typically sense conditions favourable for growth and reduce cAMP levels by activating RegA, whilst the sensor histidine phosphatases would act as stress sensors and inhibit RegA (Fig. 3). In the Dictyostelid lineage, the sensor histidine kinases acquired novel functions in cell–cell communication (Fig. 2). These novel functions subjected spore and stalk cell differentiation to strict spatiotemporal control, a defining feature of multicellular development.

### Comparative functional analysis—Genes involved in extracellular cAMP signalling

The use of cAMP as extracellular signal is thus far unique for Dictyostelia, and its role as chemoattractant in aggregation is unique for group 4 [16]. The cell surface receptor CarA, the extracellular cAMP phosphodiesterase PdsA and the adenylate cyclase AcaA are specific hallmarks of extracellular signalling, although cAMP produced by AcaA can also have second messenger roles [74]. Orthologs of *carA* were only detected in Dictyostelia, with independent gene duplications occurring in groups 1, 3 and 4 [75], [76]. In group 4, *carA* is expressed from two separate promoters. A promoter, proximal to the start codon, directs *carA* expression after aggregation, and a more distal promoter directs expression during aggregation [77]. In groups 1–3, *carA* orthologs are mainly expressed after aggregation [75], suggesting that Dictyostelia initially used secreted cAMP only after aggregation.

This was confirmed by studies showing that deletion of both copies of a duplicated *carA* gene in *P*. *pallidum* (group 2) left aggregation intact but prevented normal fruiting body morphogenesis. Deletion of *pdsA* in *P*. *pallidum* also did not affect aggregation, whilst disrupting fruiting body morphogenesis [78]. This indicates that, in contrast to aggregation, which is in *P*. *pallidum* coordinated by the chemoattractant glorin, post-aggregative cell movement is coordinated by cAMP [16], [76]. The non-hydrolysable cAMP analogue Sp-cAMPS desensitises CarA, thereby disrupting pulsatile cAMP signalling [79]. In group 4 species, Sp-cAMPS therefore effectively blocks aggregation. However, in groups 1, 2 and 3 species, Sp-cAMPS only disrupts fruiting body morphogenesis [16], [75]. Combined, these studies indicate that Dictyostelia first used pulsatile cAMP signalling to coordinate fruiting body morphogenesis. Only group 4 additionally started to use secrete cAMP to coordinate aggregation. The *pdsA* and *acaA* genes show a similar complex promoter structure as the *carA* gene with proximal promoters directing post-aggregative expression and distal promoters directing expression during aggregation [80], [81]. One evolutionary change that contributed to the use of cAMP as attractant in group 4 was therefore the addition of a distal promoter to existing cAMP signalling genes (Fig. 3).

For *carA*, the addition of the distal promoter was probably the only change needed for use of CarA during aggregation, since defective aggregation of a *D*. *discoideum cara*− mutant was fully restored by expression of a group 3 *carA* [75]. However, for a *D*. *discoideum pdsa*− mutant, expression of a group 3 *pdsA* only partially restored its aggregation-defective phenotype. Both groups 2 and 3 PdsAs have a 200-fold lower affinity for cAMP than *D*. *discoideum* PdsA. It is likely that *D*. *discoideum* PdsA requires its higher affinity to hydrolyse the lower cAMP concentrations in the aggregation field. In short, both changes in gene regulation and in gene function accompanied the novel role of cAMP in group 4 aggregation.

Apart from defective morphogenesis and disorganised stalk cell differentiation, the *P*. *pallidum car*-null fruiting bodies contained cysts instead of spores in their “spore” heads. Similar to *D*. *discoideum*, *P*. *pallidum* expresses spore coat genes in response to stimulation with extracellular cAMP, but this response was lost in the *car* null mutant [76]. As shown above, PKA activation by intracellular cAMP is sufficient for encystation, whereas spores additionally require extracellular cAMP. By deleting cAMP receptors, the *P*. *pallidum* cells reverted to the ancestral pathway of encystation.

Dictyostelids secrete most of the cAMP that they synthesise. When cells starve in isolation, secreted cAMP levels remain low and only PKA is activated, yielding cysts. However, when cells collect in aggregates, secreted cAMP accumulates to sufficient levels to activate cAMP receptors and to induce spores instead. High extracellular cAMP is therefore a signal for the aggregated state, causing cells to differentiate into spores instead of cysts. Induction of spore formation is probably the most ancestral role of secreted cAMP. The more complex mechanisms needed to produce cAMP pulses are likely to have evolved later to form the architecturally sophisticated fruiting bodies that are characteristic for the Dictyostelia (Fig. 3).

## Concluding Remarks

Aggregative multicellularity is the most common evolutionary transition from a unicellular to a multicellular life style. Many taxonomically diverse prokaryotes respond to environmental change by forming communities known as biofilms, whilst one taxon, the Myxobacteria, aggregates to form fruiting structures with spores. The latter form, also called sorocarpic multicellularity, evolved at least six times independently across most divisions of eukaryotes.

The molecular mechanisms that regulate sorocarpic development have mainly been studied in two organisms: the social amoeba *D*. *discoideum* and the myxobacterium *Myxococcus xanthus*. Despite the vast evolutionary distance between these organisms, these mechanisms have a number of features in common.

In both organisms, the formation of aggregates is initiated by starvation at high cell density, the latter being assessed by quorum sensing. Both secreted factors and direct cell–cell interactions play essential roles in coordinating the developmental programme, which for both species culminates in the differentiation of resilient spores. In both organisms, the secretion of a polysaccharide-rich matrix, otherwise known as slime, is essential for providing structural coherence, traction for cell movement and adhesion to substrata. Two-component signalling critically regulates sporulation in *Dictyostelium*, and this also appears to be the case in *Myxococcus* [82]. *Dictyostelium* uses c-di-GMP as a secreted signal to induce stalk formation. c-di-GMP induces biofilm formation in prokaryotes and roles for this molecule in extracellular matrix deposition in *Myxococcus* development are just emerging [83].

In *D*. *discoideum*, the regulation of sporulation by two-component signalling converges on controlling the levels of cAMP and thereby the activity of PKA, which is essential for spore formation. Comparative genomic analysis shows that the components of these pathways are not only conserved in all Dictyostelia but also in the amoebozoan ancestors and at least one other division of eukaryotes. Comparative functional analysis indicated that the original function for cAMP activation of PKA was to induce encystation of unicellular protozoa in response to environmental stress. Comparative studies also indicated that the manifold roles of both intracellular and secreted cAMP in regulating the developmental programme of *Dictyostelium* gradually emerged from this original role [84].

At this moment, the mechanisms controlling aggregative multicellularity in other eukaryote divisions are unknown. The similarities between *Dictyostelium* and *Myxococcus* may simply result from convergent evolution, rather than deep evolutionary conservation. However, the rapid increase in sequenced genomes for a wide variety of protists, combined with novel methods for gene manipulation, such as CRISPR-Cas9 [85] and RNA interference [86], may generate further insight in the universality of the mechanisms that control aggregative multicellularity.
